# Supplementary material for: The Use of DArTseq Technology to Identify New SNP and SilicoDArT Markers Related to the Yield-Related Traits Components in Maize
Source: Genes (Basel). 2022 May 10;13(5):848. doi: 10.3390/genes13050848 (PMC9142088; doi:10.3390/genes13050848)
Supplement: Supplementary file 1 [file genes-13-00848-s001.zip › Table S1.pdf]

**Table S1**: Mean for all lines for all analyzed features in the villages of Kobierzyce and Smolice.

| No. | Name | experimentation in Smolice | | | | | | | | | experimentation in Kobierzyce | | | | | | | | |
|---|---|---|---|---|---|---|---|---|---|---|---|---|---|---|---|---|---|---|---|
| | | cob length [cm] | cob diameter [cm] | core length [cm] | core diameter [cm] | the number of rows of grain | the number of grains in a row | mass of grain from the cob [g] | weight of one thousand grains [g] | yield [kg] | cob length [cm] | cob diameter [cm] | core length [cm] | core diameter [cm] | the number of rows of grain | the number of grains in a row | mass of grain from the cob [g] | weight of one thousand grains [g] | yield [kg] |
| 1 | **S001** | 14.3 | 4.9 | 14.0 | 2.6 | 15.3 | 22.7 | 125.0 | 398.3 | 5.0 | 15.7 | 4.6 | 15.3 | 2.3 | 16.7 | 26.7 | 159.9 | 361.2 | 6.40 |
| 2 | **S002** | 12.0 | 4.1 | 12.0 | 2.1 | 13.3 | 21.3 | 101.5 | 392.3 | 4.1 | 13.9 | 4.3 | 13.4 | 2.1 | 14.0 | 26.3 | 138.5 | 379.4 | 5.54 |
| 3 | **S003** | 14.3 | 3.9 | 14.3 | 2.3 | 15.3 | 30.3 | 102.3 | 234.0 | 4.1 | 16.8 | 4.1 | 16.6 | 2.2 | 17.3 | 34.7 | 157.0 | 262.1 | 6.28 |
| 4 | **S004** | 14.3 | 4.1 | 14.0 | 2.2 | 13.3 | 26.3 | 105.6 | 320.7 | 4.2 | 15.4 | 4.0 | 15.4 | 2.0 | 15.3 | 26.7 | 126.4 | 310.4 | 5.05 |
| 5 | **S005** | 14.7 | 3.9 | 14.7 | 2.3 | 13.3 | 30.3 | 106.3 | 285.0 | 4.3 | 16.2 | 3.9 | 16.6 | 2.2 | 14.7 | 30.7 | 122.9 | 275.8 | 4.91 |
| 6 | **S006** | 15.0 | 4.3 | 15.0 | 2.4 | 15.3 | 25.7 | 93.1 | 244.7 | 3.7 | 16.4 | 3.8 | 16.5 | 2.2 | 15.3 | 29.0 | 128.7 | 289.4 | 5.15 |
| 7 | **S007** | 13.3 | 4.2 | 13.3 | 2.6 | 17.3 | 24.0 | 101.0 | 304.3 | 4.0 | 15.7 | 4.4 | 15.6 | 2.7 | 18.0 | 26.7 | 123.3 | 256.6 | 4.93 |
| 8 | **S008** | 17.0 | 4.2 | 17.0 | 2.5 | 14.7 | 27.3 | 124.0 | 332.7 | 5.0 | 19.0 | 3.9 | 18.7 | 2.3 | 16.0 | 30.0 | 133.7 | 278.4 | 5.35 |
| 9 | **S009** | 12.7 | 4.3 | 12.7 | 2.4 | 16.0 | 25.0 | 113.3 | 322.7 | 4.5 | 14.6 | 4.1 | 14.1 | 2.3 | 16.7 | 31.7 | 134.9 | 256.5 | 5.40 |
| 10 | **S010** | 14.7 | 4.2 | 14.3 | 2.0 | 15.3 | 28.3 | 113.8 | 287.7 | 4.6 | 15.7 | 3.8 | 15.4 | 2.0 | 16.7 | 32.7 | 146.7 | 270.0 | 5.87 |
| 11 | **S011** | 13.3 | 4.3 | 13.2 | 2.6 | 16.0 | 23.7 | 97.5 | 290.0 | 3.9 | 14.8 | 4.3 | 15.1 | 2.3 | 16.7 | 26.7 | 116.5 | 266.2 | 4.66 |
| 12 | **S012** | 14.7 | 4.3 | 14.2 | 1.2 | 14.0 | 28.7 | 124.6 | 333.3 | 5.0 | 17.0 | 4.1 | 17.0 | 2.1 | 14.7 | 33.0 | 165.0 | 342.4 | 6.60 |
| 13 | **S013** | 17.3 | 3.9 | 16.7 | 1.2 | 14.0 | 29.3 | 97.5 | 260.0 | 3.9 | 18.2 | 3.7 | 17.5 | 2.2 | 16.0 | 30.0 | 116.9 | 244.1 | 4.67 |
| 14 | **S014** | 16.0 | 4.2 | 16.3 | 1.1 | 14.7 | 27.3 | 86.3 | 229.3 | 3.5 | 17.3 | 4.2 | 17.3 | 2.5 | 15.3 | 34.0 | 150.2 | 287.8 | 6.01 |
| 15 | **S015** | 12.7 | 3.9 | 12.7 | 0.9 | 10.7 | 17.0 | 55.7 | 319.3 | 2.2 | 14.0 | 4.0 | 14.1 | 2.2 | 12.7 | 26.3 | 106.6 | 322.2 | 4.27 |
| 16 | **S016** | 13.0 | 4.4 | 12.7 | 0.9 | 16.7 | 29.0 | 99.0 | 231.7 | 4.0 | 13.7 | 4.1 | 13.5 | 2.2 | 16.0 | 30.0 | 109.7 | 231.9 | 4.39 |
| 17 | **S017** | 14.7 | 4.7 | 14.3 | 1.0 | 17.3 | 26.0 | 106.9 | 301.7 | 4.3 | 15.3 | 4.0 | 15.0 | 2.0 | 18.0 | 30.7 | 121.8 | 221.2 | 4.87 |
| 18 | **S018** | 15.0 | 4.6 | 14.8 | 1.0 | 20.7 | 28.0 | 95.9 | 204.7 | 3.8 | 16.5 | 4.7 | 16.1 | 2.6 | 21.3 | 33.3 | 145.1 | 201.6 | 5.80 |
| 19 | **S019** | 12.3 | 4.1 | 12.0 | 1.0 | 16.0 | 24.0 | 79.9 | 268.0 | 3.2 | 14.7 | 4.1 | 14.7 | 2.2 | 15.3 | 27.0 | 112.8 | 271.4 | 4.51 |
| 20 | **S020** | 13.3 | 3.5 | 13.3 | 0.8 | 13.3 | 23.3 | 61.5 | 262.7 | 2.5 | 16.0 | 3.8 | 15.5 | 2.1 | 14.7 | 30.0 | 122.5 | 281.2 | 4.90 |
| 21 | **S021** | 13.0 | 3.7 | 12.7 | 0.8 | 11.3 | 20.7 | 76.0 | 368.0 | 3.0 | 14.0 | 3.5 | 13.7 | 1.8 | 12.0 | 22.7 | 94.8 | 346.8 | 3.79 |
| 22 | **S022** | 13.3 | 4.4 | 13.2 | 2.4 | 17.3 | 24.3 | 114.9 | 325.3 | 4.6 | 14.0 | 4.4 | 13.5 | 2.4 | 18.0 | 25.0 | 123.3 | 273.6 | 4.93 |
| 23 | **S023** | 14.7 | 3.7 | 14.3 | 2.4 | 12.0 | 24.7 | 76.5 | 328.7 | 3.1 | 14.9 | 3.2 | 15.3 | 2.0 | 12.0 | 26.7 | 69.7 | 218.7 | 2.79 |
| 24 | **S024** | 16.3 | 3.3 | 16.3 | 2.0 | 12.0 | 28.3 | 78.2 | 274.7 | 3.1 | 17.2 | 3.2 | 17.2 | 1.7 | 14.0 | 29.3 | 100.6 | 244.3 | 4.02 |
| 25 | **S025** | 13.3 | 4.0 | 13.3 | 2.3 | 14.0 | 26.0 | 95.0 | 313.7 | 3.8 | 13.7 | 4.0 | 13.2 | 2.1 | 16.0 | 26.7 | 112.0 | 261.6 | 4.48 |
| 26 | **S026** | 14.3 | 3.9 | 14.0 | 2.0 | 16.0 | 29.0 | 102.5 | 252.0 | 4.1 | 14.8 | 3.3 | 14.5 | 1.6 | 14.7 | 29.3 | 105.3 | 243.0 | 4.21 |
| 27 | **S027** | 13.7 | 3.3 | 14.0 | 1.9 | 12.7 | 24.7 | 58.3 | 243.3 | 2.3 | 15.1 | 3.1 | 14.8 | 1.8 | 12.0 | 24.0 | 71.2 | 245.8 | 2.85 |
| 28 | **S028** | 13.0 | 3.5 | 14.0 | 2.0 | 13.3 | 24.3 | 70.9 | 283.3 | 2.8 | 16.2 | 3.9 | 16.7 | 1.9 | 16.0 | 31.7 | 133.0 | 263.4 | 5.32 |
| 29 | **S029** | 11.7 | 3.7 | 12.0 | 1.9 | 12.7 | 21.7 | 78.1 | 337.0 | 3.1 | 15.3 | 3.7 | 14.7 | 1.7 | 13.3 | 28.3 | 119.0 | 314.2 | 4.76 |
| 30 | **S030** | 13.7 | 4.2 | 14.3 | 2.3 | 12.7 | 29.7 | 112.0 | 327.0 | 4.5 | 16.5 | 4.0 | 16.3 | 2.1 | 14.7 | 26.7 | 121.2 | 316.2 | 4.85 |
| 31 | **S031** | 13.0 | 4.3 | 13.3 | 2.4 | 15.3 | 25.7 | 111.2 | 312.7 | 4.4 | 14.0 | 4.1 | 13.8 | 2.0 | 14.0 | 24.3 | 98.0 | 284.4 | 3.92 |
| 32 | **S032** | 12.0 | 3.9 | 11.8 | 2.5 | 14.7 | 20.7 | 83.0 | 314.7 | 3.3 | 12.8 | 4.3 | 13.0 | 2.4 | 18.0 | 23.7 | 115.1 | 270.2 | 4.60 |

| | | | | | | | | | | | | | | | | | | | |
|---|---|---|---|---|---|---|---|---|---|---|---|---|---|---|---|---|---|---|---|
| 33 | **S033** | 12.0 | 4.0 | 12.7 | 2.5 | 14.7 | 28.7 | 81.8 | 218.0 | 3.3 | 14.8 | 4.4 | 14.8 | 2.6 | 16.7 | 29.3 | 128.5 | 264.3 | 5.14 |
| 34 | **S034** | 14.0 | 4.5 | 13.5 | 2.4 | 18.7 | 27.7 | 117.2 | 267.0 | 4.7 | 15.5 | 4.7 | 15.6 | 2.2 | 21.3 | 29.3 | 125.6 | 195.5 | 5.02 |
| 35 | **S035** | 12.3 | 4.6 | 12.2 | 2.7 | 19.3 | 25.3 | 101.7 | 246.0 | 4.1 | 15.7 | 4.9 | 15.7 | 2.6 | 22.0 | 26.7 | 126.0 | 213.9 | 5.04 |
| 36 | **S036** | 12.3 | 4.0 | 12.3 | 2.3 | 14.7 | 30.0 | 99.3 | 240.3 | 4.0 | 13.5 | 3.9 | 13.0 | 2.0 | 15.3 | 28.0 | 99.4 | 234.6 | 3.98 |
| 37 | **S037** | 13.0 | 4.5 | 13.5 | 2.4 | 15.3 | 24.0 | 114.6 | 311.7 | 4.6 | 16.2 | 4.3 | 16.1 | 2.5 | 17.3 | 30.7 | 139.1 | 262.1 | 5.56 |
| 38 | **S038** | 11.7 | 4.2 | 12.5 | 2.3 | 15.3 | 23.0 | 87.8 | 341.0 | 3.5 | 16.8 | 4.1 | 16.8 | 2.2 | 15.3 | 30.0 | 122.6 | 266.0 | 4.91 |
| 39 | **S039** | 12.7 | 4.6 | 12.8 | 2.6 | 14.7 | 27.0 | 111.1 | 322.7 | 4.4 | 14.3 | 4.8 | 14.4 | 2.7 | 17.3 | 26.0 | 146.2 | 325.1 | 5.85 |
| 40 | **S040** | 11.3 | 4.5 | 12.2 | 2.5 | 16.0 | 19.7 | 95.4 | 377.3 | 3.8 | 16.7 | 4.5 | 16.5 | 2.4 | 15.3 | 29.3 | 140.1 | 311.7 | 5.60 |
| 41 | **S041** | 15.3 | 4.5 | 15.5 | 2.6 | 16.7 | 32.0 | 119.9 | 250.3 | 4.8 | 16.4 | 4.5 | 15.9 | 2.5 | 20.0 | 30.0 | 130.5 | 218.1 | 5.22 |
| 42 | **S042** | 14.3 | 3.4 | 15.3 | 2.0 | 12.7 | 26.7 | 81.5 | 296.7 | 3.3 | 16.5 | 3.6 | 16.4 | 1.9 | 14.7 | 29.0 | 99.9 | 225.1 | 4.00 |
| 43 | **S043** | 11.0 | 4.2 | 11.3 | 2.1 | 14.7 | 22.7 | 88.1 | 325.3 | 3.5 | 12.0 | 3.8 | 12.5 | 1.9 | 13.3 | 23.3 | 88.0 | 282.4 | 3.52 |
| 44 | **S044** | 10.0 | 4.6 | 10.7 | 2.6 | 17.3 | 19.0 | 80.7 | 297.7 | 3.2 | 14.0 | 4.8 | 14.1 | 3.1 | 18.0 | 22.0 | 109.7 | 277.3 | 4.39 |
| 45 | **S045** | 13.3 | 4.4 | 14.0 | 2.4 | 16.7 | 28.7 | 116.0 | 298.0 | 4.6 | 17.3 | 4.2 | 16.8 | 2.2 | 18.0 | 36.0 | 136.4 | 210.5 | 5.46 |
| 46 | **S046** | 13.0 | 3.6 | 13.3 | 2.2 | 14.0 | 23.3 | 70.4 | 275.7 | 2.8 | 15.4 | 3.4 | 15.5 | 2.1 | 12.0 | 24.0 | 83.0 | 286.9 | 3.32 |
| 47 | **S047** | 15.3 | 4.4 | 15.3 | 2.3 | 14.0 | 28.7 | 137.2 | 324.7 | 5.5 | 16.7 | 4.2 | 16.3 | 2.2 | 15.3 | 29.3 | 141.5 | 317.1 | 5.66 |
| 48 | **S048** | 15.0 | 4.3 | 14.8 | 2.3 | 14.7 | 29.7 | 123.5 | 333.3 | 4.9 | 16.7 | 4.1 | 16.3 | 2.2 | 14.0 | 28.3 | 133.8 | 339.0 | 5.35 |
| 49 | **S049** | 14.3 | 3.7 | 14.7 | 2.1 | 13.3 | 30.3 | 85.9 | 253.3 | 3.4 | 13.8 | 3.5 | 13.5 | 2.0 | 14.0 | 26.0 | 87.9 | 246.8 | 3.52 |
| 50 | **S050** | 13.7 | 3.7 | 14.0 | 2.0 | 16.0 | 28.3 | 87.7 | 216.3 | 3.5 | 16.5 | 3.6 | 16.2 | 1.8 | 16.7 | 34.0 | 111.7 | 199.2 | 4.47 |
| 51 | **S051** | 17.3 | 4.3 | 17.5 | 2.3 | 13.3 | 31.7 | 142.8 | 381.7 | 5.7 | 19.3 | 3.8 | 18.8 | 2.1 | 13.3 | 29.0 | 74.0 | 188.0 | 2.96 |
| 52 | **S052** | 14.0 | 4.5 | 14.2 | 2.7 | 17.3 | 28.0 | 119.4 | 254.3 | 4.8 | 15.6 | 4.2 | 15.1 | 2.8 | 18.0 | 27.7 | 114.4 | 229.7 | 4.57 |
| 53 | **S053** | 14.0 | 4.6 | 14.2 | 2.8 | 20.0 | 28.7 | 126.8 | 265.3 | 5.1 | 15.0 | 4.5 | 14.7 | 2.9 | 19.3 | 29.0 | 144.7 | 259.2 | 5.79 |
| 54 | **S054** | 12.0 | 4.2 | 12.2 | 2.4 | 16.7 | 23.7 | 95.9 | 277.0 | 3.8 | 13.9 | 4.2 | 13.9 | 2.2 | 19.3 | 25.3 | 115.8 | 237.4 | 4.63 |
| 55 | **S055** | 16.0 | 4.0 | 16.2 | 2.2 | 13.3 | 29.3 | 115.6 | 335.7 | 4.6 | 16.2 | 3.7 | 16.1 | 2.0 | 13.3 | 28.0 | 113.4 | 304.9 | 4.53 |
| 56 | **S056** | 15.3 | 4.0 | 16.0 | 2.3 | 14.0 | 29.3 | 107.0 | 316.7 | 4.3 | 19.8 | 4.2 | 19.6 | 2.2 | 16.0 | 31.3 | 148.1 | 295.2 | 5.92 |
| 57 | **S057** | 17.3 | 4.2 | 17.3 | 2.1 | 14.7 | 33.3 | 139.1 | 304.7 | 5.6 | 19.2 | 4.0 | 19.4 | 2.0 | 16.0 | 34.0 | 150.3 | 281.5 | 6.01 |
| 58 | **S058** | 16.0 | 3.9 | 17.7 | 2.1 | 14.0 | 29.0 | 107.0 | 298.0 | 4.3 | 21.0 | 3.7 | 21.0 | 2.0 | 16.0 | 32.0 | 118.9 | 232.3 | 4.76 |
| 59 | **S059** | 14.0 | 5.4 | 14.0 | 2.9 | 21.3 | 25.0 | 145.9 | 342.3 | 5.8 | 17.3 | 5.4 | 16.7 | 3.2 | 19.3 | 24.0 | 154.1 | 327.1 | 6.16 |
| 60 | **S060** | 15.7 | 4.0 | 15.7 | 2.4 | 12.7 | 28.3 | 103.6 | 311.0 | 4.1 | 18.0 | 3.9 | 19.0 | 2.4 | 12.7 | 31.0 | 109.6 | 280.2 | 4.39 |
| 61 | **S061** | 13.3 | 4.5 | 14.3 | 2.6 | 19.3 | 28.0 | 116.1 | 256.7 | 4.6 | 16.3 | 4.9 | 16.3 | 2.5 | 21.3 | 28.7 | 147.6 | 239.1 | 5.90 |
| 62 | **S062** | 14.3 | 4.7 | 14.0 | 2.8 | 20.0 | 29.0 | 131.1 | 262.0 | 5.2 | 16.5 | 4.9 | 16.1 | 2.5 | 22.0 | 29.3 | 159.1 | 246.6 | 6.37 |
| 63 | **S063** | 14.7 | 4.2 | 15.3 | 2.5 | 14.7 | 24.7 | 109.9 | 332.3 | 4.4 | 18.3 | 4.3 | 17.8 | 2.4 | 16.7 | 28.7 | 124.6 | 262.0 | 4.98 |
| 64 | **S064** | 12.0 | 4.1 | 11.3 | 2.4 | 12.7 | 25.0 | 96.0 | 332.3 | 3.8 | 12.8 | 4.0 | 12.2 | 2.2 | 14.0 | 25.7 | 107.6 | 300.1 | 4.30 |
| 65 | **S065** | 15.7 | 4.6 | 15.7 | 2.8 | 18.7 | 26.3 | 140.4 | 327.3 | 5.6 | 17.9 | 4.6 | 17.7 | 2.8 | 18.0 | 28.7 | 133.6 | 259.5 | 5.34 |
| 66 | **S066** | 13.0 | 4.4 | 13.3 | 2.6 | 16.7 | 23.3 | 102.3 | 315.7 | 4.1 | 15.3 | 4.3 | 15.0 | 2.4 | 17.3 | 26.0 | 122.0 | 292.1 | 4.90 |
| 67 | **S067** | 14.7 | 3.9 | 15.7 | 2.3 | 14.0 | 25.0 | 86.0 | 328.3 | 3.4 | 17.0 | 3.8 | 16.8 | 2.0 | 14.7 | 27.7 | 106.2 | 304.6 | 4.20 |
| 68 | **S068** | 14.7 | 4.1 | 14.7 | 2.4 | 15.3 | 24.3 | 99.8 | 305.0 | 4.0 | 17.0 | 4.0 | 16.6 | 2.1 | 16.0 | 27.0 | 120.1 | 281.5 | 4.80 |
| 69 | **S069** | 13.3 | 4.1 | 13.3 | 2.4 | 14.7 | 24.3 | 91.2 | 306.7 | 3.6 | 15.7 | 4.1 | 15.4 | 2.2 | 16.0 | 30.7 | 128.5 | 262.3 | 5.14 |
| 70 | **S070** | 12.0 | 4.4 | 13.3 | 2.5 | 16.7 | 26.7 | 95.2 | 251.0 | 3.8 | 15.7 | 4.1 | 15.6 | 2.3 | 17.3 | 31.3 | 116.0 | 213.8 | 4.64 |
| 71 | **S071** | 13.7 | 3.4 | 13.7 | 2.0 | 12.0 | 29.3 | 71.2 | 237.0 | 2.8 | 14.5 | 2.9 | 14.4 | 1.6 | 12.0 | 28.0 | 66.3 | 196.4 | 2.65 |
| 72 | **S072** | 13.7 | 4.0 | 14.7 | 2.2 | 15.3 | 29.7 | 102.1 | 255.7 | 4.1 | 17.0 | 3.7 | 16.5 | 1.9 | 14.7 | 30.7 | 104.7 | 237.1 | 4.19 |

| | | | | | | | | | | | | | | | | | | | |
|---|---|---|---|---|---|---|---|---|---|---|---|---|---|---|---|---|---|---|---|
| 73 | **S073** | 15.0 | 3.7 | 15.3 | 2.4 | 16.7 | 30.7 | 73.4 | 176.7 | 2.9 | 18.0 | 3.5 | 18.4 | 2.0 | 16.0 | 34.3 | 99.3 | 180.6 | 3.97 |
| 74 | **S074** | 15.0 | 3.5 | 16.0 | 2.0 | 14.7 | 27.3 | 69.5 | 188.0 | 2.8 | 17.5 | 3.3 | 17.4 | 1.7 | 14.7 | 30.0 | 91.1 | 205.3 | 3.64 |
| 75 | **S075** | 14.0 | 3.6 | 14.7 | 2.1 | 14.0 | 22.7 | 65.3 | 255.3 | 2.6 | 17.5 | 3.5 | 17.5 | 1.9 | 14.0 | 27.7 | 94.7 | 244.2 | 3.79 |
| 76 | **S076** | 16.0 | 3.6 | 16.0 | 1.9 | 12.7 | 31.7 | 89.1 | 250.3 | 3.6 | 16.8 | 3.5 | 18.5 | 1.8 | 15.3 | 29.3 | 86.5 | 192.9 | 3.46 |
| 77 | **S077** | 15.0 | 3.6 | 15.0 | 2.2 | 14.0 | 28.3 | 78.4 | 233.7 | 3.1 | 16.4 | 3.5 | 16.2 | 2.0 | 15.3 | 28.3 | 94.2 | 215.5 | 3.77 |
| 78 | **S078** | 13.0 | 3.7 | 12.7 | 2.0 | 11.3 | 11.3 | 73.9 | 312.3 | 3.0 | 17.2 | 3.6 | 17.3 | 1.9 | 12.7 | 25.3 | 97.7 | 304.8 | 3.91 |
| 79 | **S079** | 11.3 | 3.8 | 12.0 | 2.0 | 16.7 | 16.7 | 79.9 | 276.3 | 3.2 | 11.8 | 3.7 | 11.4 | 2.0 | 16.7 | 21.0 | 78.7 | 227.1 | 3.15 |
| 80 | **S080** | 13.0 | 4.2 | 13.0 | 2.3 | 18.7 | 18.7 | 110.0 | 286.0 | 4.4 | 15.3 | 4.0 | 14.8 | 2.1 | 20.7 | 30.0 | 122.1 | 197.3 | 4.88 |
| 81 | **S081** | 10.3 | 4.2 | 13.0 | 2.4 | 14.0 | 14.0 | 77.6 | 378.7 | 3.1 | 15.5 | 3.8 | 15.8 | 2.0 | 14.0 | 20.7 | 96.4 | 333.2 | 3.86 |
| 82 | **S082** | 12.0 | 3.8 | 12.0 | 2.2 | 16.7 | 16.7 | 63.3 | 243.7 | 2.5 | 17.3 | 4.1 | 17.9 | 2.2 | 18.7 | 28.3 | 133.0 | 254.4 | 5.32 |
| 83 | **S083** | 13.3 | 4.5 | 13.3 | 2.6 | 14.7 | 14.7 | 113.5 | 339.7 | 4.5 | 13.9 | 4.1 | 14.0 | 2.3 | 16.0 | 24.7 | 104.6 | 266.9 | 4.18 |
| 84 | **S084** | 12.3 | 4.5 | 13.3 | 2.8 | 16.7 | 16.7 | 85.8 | 387.3 | 3.4 | 15.2 | 4.6 | 15.2 | 2.8 | 17.3 | 21.3 | 109.9 | 298.8 | 4.40 |
| 85 | **S085** | 10.7 | 3.8 | 11.3 | 2.3 | 12.7 | 12.7 | 68.0 | 343.0 | 2.7 | 13.7 | 4.2 | 14.0 | 2.1 | 15.3 | 24.0 | 105.9 | 285.8 | 4.24 |
| 86 | **S086** | 12.0 | 3.8 | 12.0 | 2.3 | 13.3 | 13.3 | 71.3 | 309.3 | 2.9 | 15.0 | 3.8 | 14.8 | 2.3 | 14.7 | 25.3 | 103.7 | 279.5 | 4.15 |
| 87 | **S087** | 11.7 | 4.1 | 11.7 | 2.4 | 14.0 | 14.0 | 74.7 | 276.3 | 3.0 | 13.6 | 4.0 | 14.1 | 2.1 | 13.3 | 21.0 | 75.8 | 274.3 | 3.03 |
| 88 | **S088** | 14.3 | 4.2 | 15.3 | 2.5 | 15.3 | 15.3 | 108.4 | 297.3 | 4.3 | 16.9 | 4.1 | 17.5 | 2.1 | 15.3 | 32.7 | 121.8 | 244.2 | 4.87 |
| 89 | **S089** | 13.7 | 4.0 | 14.3 | 2.4 | 14.7 | 22.7 | 85.3 | 286.3 | 3.4 | 16.7 | 3.7 | 16.8 | 2.0 | 14.7 | 25.3 | 98.5 | 264.1 | 3.94 |
| 90 | **S090** | 11.0 | 3.7 | 11.0 | 2.2 | 14.0 | 23.3 | 77.0 | 230.0 | 3.1 | 12.8 | 3.5 | 12.6 | 1.8 | 14.0 | 26.3 | 95.6 | 261.7 | 3.82 |
| 91 | **S091** | 12.0 | 3.6 | 12.7 | 2.2 | 15.3 | 25.7 | 58.7 | 189.0 | 2.3 | 14.2 | 3.5 | 14.4 | 2.0 | 16.0 | 31.3 | 81.6 | 162.7 | 3.26 |
| 92 | **S092** | 10.7 | 4.6 | 10.7 | 2.6 | 15.3 | 21.3 | 92.8 | 317.3 | 3.7 | 15.1 | 4.4 | 14.4 | 2.7 | 18.7 | 24.7 | 114.1 | 248.2 | 4.56 |
| 93 | **S093** | 13.0 | 4.0 | 13.0 | 2.4 | 14.7 | 23.7 | 83.7 | 255.3 | 3.3 | 13.0 | 3.7 | 13.0 | 2.1 | 16.0 | 25.0 | 96.4 | 241.3 | 3.85 |
| 94 | **S094** | 12.7 | 4.3 | 13.0 | 2.6 | 16.7 | 24.3 | 102.2 | 294.0 | 4.1 | 17.5 | 4.3 | 17.8 | 2.4 | 19.3 | 28.7 | 136.0 | 242.6 | 5.44 |
| 95 | **S095** | 13.0 | 4.5 | 13.0 | 2.5 | 18.0 | 24.7 | 118.3 | 323.0 | 4.7 | 15.3 | 4.7 | 15.6 | 2.5 | 18.7 | 26.3 | 133.4 | 271.8 | 5.34 |
| 96 | **S096** | 13.7 | 4.4 | 13.7 | 2.5 | 16.0 | 26.0 | 108.9 | 298.7 | 4.4 | 15.2 | 3.9 | 15.1 | 2.2 | 16.7 | 29.3 | 122.2 | 250.6 | 4.89 |
| 97 | **S097** | 12.7 | 3.8 | 12.7 | 2.1 | 14.0 | 28.3 | 83.8 | 210.0 | 3.4 | 13.8 | 3.5 | 13.5 | 1.7 | 16.0 | 30.3 | 101.1 | 210.1 | 4.04 |
| 98 | **S098** | 12.3 | 3.8 | 12.7 | 2.1 | 14.0 | 26.7 | 81.7 | 218.3 | 3.3 | 15.4 | 3.5 | 15.5 | 1.8 | 14.7 | 30.7 | 101.9 | 227.6 | 4.08 |
| 99 | **S099** | 12.3 | 3.6 | 12.3 | 2.1 | 14.7 | 26.3 | 62.4 | 194.0 | 2.5 | 14.7 | 3.3 | 14.6 | 1.6 | 12.7 | 30.3 | 76.9 | 196.9 | 3.08 |
| 100 | **S100** | 10.3 | 3.7 | 10.7 | 2.0 | 16.7 | 28.0 | 70.4 | 172.3 | 2.8 | 10.2 | 3.3 | 10.3 | 1.5 | 15.3 | 21.0 | 56.2 | 174.1 | 2.25 |
| 101 | **S101** | 15.0 | 4.2 | 15.0 | 2.3 | 12.7 | 27.3 | 112.4 | 365.3 | 4.5 | 17.5 | 4.0 | 17.8 | 2.3 | 14.0 | 24.7 | 113.5 | 329.9 | 4.54 |
| 102 | **S102** | 13.3 | 3.8 | 13.3 | 2.0 | 14.0 | 25.0 | 93.4 | 300.3 | 3.7 | 15.8 | 3.5 | 15.8 | 1.8 | 14.7 | 27.3 | 114.7 | 289.6 | 4.59 |
| 103 | **S103** | 13.0 | 3.9 | 13.0 | 2.1 | 15.3 | 29.7 | 97.2 | 230.0 | 3.9 | 15.0 | 3.8 | 15.1 | 1.9 | 15.3 | 33.0 | 110.7 | 219.4 | 4.43 |
| 104 | **S104** | 13.7 | 3.9 | 13.7 | 2.2 | 13.3 | 29.0 | 97.6 | 278.0 | 3.9 | 15.3 | 3.7 | 15.3 | 1.9 | 14.0 | 30.7 | 117.3 | 279.0 | 4.69 |
| 105 | **S105** | 11.0 | 3.8 | 11.7 | 2.2 | 13.3 | 20.7 | 55.2 | 305.3 | 2.2 | 14.7 | 3.9 | 15.1 | 2.1 | 14.7 | 24.0 | 93.0 | 266.1 | 3.72 |
| 106 | **S106** | 13.7 | 4.2 | 13.7 | 2.4 | 14.7 | 30.3 | 103.5 | 263.3 | 4.1 | 15.7 | 4.0 | 15.6 | 2.2 | 15.3 | 31.7 | 120.1 | 248.6 | 4.80 |
| 107 | **S107** | 10.7 | 3.8 | 11.0 | 2.2 | 12.0 | 22.3 | 67.9 | 300.3 | 2.7 | 13.2 | 3.6 | 13.1 | 1.9 | 12.7 | 25.3 | 94.3 | 295.4 | 3.77 |
| 108 | **S108** | 10.7 | 4.2 | 11.0 | 2.4 | 14.0 | 23.3 | 72.3 | 253.3 | 2.9 | 16.2 | 4.5 | 16.0 | 2.6 | 16.0 | 27.7 | 126.5 | 286.7 | 5.06 |
| 109 | **S109** | 11.7 | 4.1 | 12.3 | 2.3 | 14.0 | 22.0 | 81.8 | 329.3 | 3.3 | 18.1 | 4.1 | 18.0 | 2.0 | 17.3 | 29.7 | 144.5 | 281.9 | 5.78 |
| 110 | **S110** | 14.7 | 4.2 | 14.7 | 2.2 | 15.3 | 27.3 | 115.6 | 306.3 | 4.6 | 15.5 | 3.8 | 15.2 | 1.9 | 14.0 | 27.3 | 122.8 | 324.6 | 4.91 |
| 111 | **S111** | 13.0 | 3.9 | 13.0 | 2.1 | 14.7 | 26.3 | 82.2 | 298.0 | 3.3 | 15.2 | 3.5 | 14.7 | 1.9 | 14.0 | 25.7 | 101.6 | 284.5 | 4.06 |
| 112 | **S112** | 17.0 | 3.8 | 17.0 | 2.0 | 16.0 | 33.3 | 117.8 | 283.0 | 4.7 | 18.1 | 3.5 | 18.3 | 1.6 | 16.0 | 31.7 | 130.5 | 259.5 | 5.22 |

| | | | | | | | | | | | | | | | | | | | |
|---|---|---|---|---|---|---|---|---|---|---|---|---|---|---|---|---|---|---|---|
| 113 | **S113** | 14.0 | 4.0 | 14.0 | 2.3 | 14.7 | 33.3 | 94.9 | 215.3 | 3.8 | 15.3 | 3.5 | 15.3 | 1.8 | 14.7 | 30.3 | 101.2 | 227.5 | 4.05 |
| 114 | **S114** | 11.0 | 4.7 | 11.0 | 2.8 | 18.7 | 24.0 | 91.7 | 224.3 | 3.7 | 12.7 | 4.6 | 12.2 | 2.6 | 18.0 | 25.3 | 109.7 | 239.0 | 4.39 |
| 115 | **S115** | 15.0 | 4.2 | 15.0 | 2.4 | 13.3 | 30.7 | 106.6 | 272.3 | 4.3 | 14.6 | 3.7 | 14.1 | 2.0 | 12.7 | 29.3 | 104.0 | 277.8 | 4.16 |
| 116 | **S116** | 12.0 | 3.8 | 12.0 | 2.3 | 13.3 | 23.3 | 78.3 | 313.7 | 3.1 | 13.8 | 3.6 | 13.7 | 1.9 | 12.7 | 24.3 | 95.2 | 308.9 | 3.81 |
| 117 | **S117** | 12.3 | 4.2 | 12.3 | 2.4 | 15.3 | 24.7 | 96.2 | 251.7 | 3.8 | 14.5 | 4.3 | 14.0 | 2.2 | 15.3 | 28.0 | 129.7 | 304.2 | 5.19 |
| 118 | **S118** | 13.0 | 4.4 | 13.3 | 2.6 | 14.7 | 23.0 | 84.7 | 305.7 | 3.4 | 13.9 | 4.2 | 13.6 | 2.4 | 16.0 | 23.3 | 112.9 | 304.2 | 4.51 |
| 119 | **S119** | 14.7 | 3.7 | 16.0 | 2.3 | 12.7 | 25.3 | 71.8 | 254.0 | 2.9 | 17.6 | 3.8 | 17.6 | 2.1 | 12.7 | 32.7 | 124.2 | 299.9 | 4.97 |
| 120 | **S120** | 13.7 | 4.3 | 14.7 | 2.6 | 15.3 | 26.0 | 100.4 | 295.3 | 4.0 | 17.0 | 4.2 | 17.3 | 2.5 | 14.0 | 31.7 | 129.6 | 292.9 | 5.18 |
| 121 | **S121** | 13.0 | 4.2 | 13.7 | 2.5 | 12.7 | 28.0 | 88.8 | 288.0 | 3.6 | 14.9 | 4.1 | 14.6 | 2.3 | 16.0 | 30.7 | 136.1 | 277.6 | 5.44 |
| 122 | **S122** | 13.0 | 4.3 | 13.0 | 2.4 | 14.0 | 25.0 | 92.4 | 284.3 | 3.7 | 14.2 | 4.1 | 14.3 | 2.2 | 15.3 | 25.7 | 120.9 | 308.2 | 4.84 |
| 123 | **S123** | 16.3 | 3.9 | 17.0 | 2.1 | 14.7 | 33.0 | 124.3 | 280.0 | 5.0 | 17.5 | 3.8 | 17.2 | 2.0 | 16.7 | 35.3 | 137.6 | 236.4 | 5.51 |
| 124 | **S124** | 13.7 | 4.2 | 14.3 | 2.2 | 15.3 | 24.7 | 103.3 | 318.3 | 4.1 | 16.0 | 4.1 | 16.0 | 2.1 | 15.3 | 24.7 | 111.8 | 294.7 | 4.47 |
| 125 | **S125** | 13.7 | 4.0 | 14.0 | 2.3 | 12.7 | 21.3 | 78.7 | 313.7 | 3.1 | 16.7 | 3.8 | 16.5 | 2.1 | 12.7 | 27.0 | 105.3 | 308.9 | 4.21 |
| 126 | **S126** | 13.3 | 3.9 | 14.0 | 2.3 | 16.0 | 22.7 | 91.0 | 285.3 | 3.6 | 16.5 | 3.6 | 16.6 | 2.0 | 15.3 | 26.7 | 89.7 | 219.9 | 3.59 |
| 127 | **S127** | 12.7 | 4.2 | 13.3 | 2.5 | 14.7 | 24.3 | 96.9 | 322.3 | 3.9 | 18.2 | 4.3 | 18.1 | 2.5 | 16.7 | 30.7 | 150.9 | 299.7 | 6.04 |
| 128 | **S128** | 12.7 | 4.3 | 12.7 | 2.6 | 18.7 | 23.7 | 90.1 | 268.3 | 3.6 | 13.5 | 4.2 | 13.7 | 2.5 | 17.3 | 21.0 | 92.3 | 249.2 | 3.69 |
| 129 | **S129** | 13.3 | 4.3 | 13.3 | 2.4 | 16.0 | 30.7 | 103.4 | 229.0 | 4.1 | 15.6 | 4.1 | 15.2 | 2.2 | 17.3 | 32.7 | 134.1 | 237.5 | 5.36 |
| 130 | **S130** | 11.7 | 3.8 | 11.7 | 2.2 | 14.0 | 22.0 | 72.0 | 299.7 | 2.9 | 15.2 | 4.0 | 15.1 | 2.2 | 15.3 | 26.3 | 122.7 | 306.0 | 4.91 |
| 131 | **S131** | 13.0 | 4.0 | 13.0 | 2.3 | 14.7 | 29.3 | 100.4 | 287.0 | 4.0 | 14.5 | 3.9 | 14.7 | 2.2 | 17.3 | 29.3 | 113.5 | 224.8 | 4.54 |
| 132 | **S132** | 15.3 | 4.1 | 15.7 | 2.4 | 13.3 | 24.7 | 116.1 | 354.3 | 4.6 | 18.0 | 4.0 | 18.0 | 2.1 | 14.0 | 28.7 | 133.9 | 332.4 | 5.36 |
| 133 | **S133** | 12.0 | 3.7 | 12.0 | 2.4 | 15.3 | 23.0 | 50.3 | 178.7 | 2.0 | 12.6 | 3.3 | 12.4 | 1.9 | 16.0 | 23.7 | 57.6 | 151.8 | 2.30 |
| 134 | **S134** | 10.7 | 4.2 | 10.7 | 2.3 | 17.3 | 23.3 | 81.9 | 255.7 | 3.3 | 12.0 | 4.0 | 11.7 | 2.3 | 18.0 | 23.7 | 99.0 | 232.1 | 3.96 |
| 135 | **S135** | 12.0 | 3.8 | 12.0 | 2.1 | 14.0 | 21.0 | 72.2 | 290.7 | 2.9 | 13.7 | 3.9 | 13.3 | 2.0 | 16.0 | 24.0 | 93.4 | 247.6 | 3.74 |
| 136 | **S136** | 11.3 | 4.1 | 11.3 | 2.3 | 14.7 | 20.0 | 75.5 | 323.0 | 3.0 | 14.3 | 3.9 | 14.5 | 2.3 | 13.3 | 22.3 | 99.3 | 325.9 | 3.97 |
| 137 | **S137** | 12.0 | 4.2 | 12.3 | 2.4 | 18.7 | 22.3 | 90.0 | 243.0 | 3.6 | 15.0 | 4.1 | 14.8 | 2.3 | 20.7 | 25.3 | 116.1 | 223.0 | 4.64 |
| 138 | **S138** | 11.3 | 4.2 | 11.3 | 2.4 | 19.3 | 21.7 | 82.4 | 245.7 | 3.3 | 14.9 | 4.3 | 14.5 | 2.4 | 20.0 | 29.3 | 147.3 | 252.4 | 5.89 |
| 139 | **S139** | 12.3 | 3.9 | 12.5 | 2.2 | 14.0 | 27.3 | 91.4 | 266.0 | 3.7 | 13.9 | 3.8 | 13.5 | 1.8 | 13.3 | 30.7 | 113.3 | 279.9 | 4.53 |
| 140 | **S140** | 13.0 | 4.1 | 13.0 | 2.2 | 14.0 | 2.2 | 76.3 | 384.0 | 3.1 | 15.9 | 3.7 | 15.8 | 2.1 | 14.7 | 22.0 | 80.4 | 245.9 | 3.22 |
| 141 | **S141** | 12.3 | 4.0 | 15.7 | 2.3 | 13.3 | 27.3 | 83.3 | 259.3 | 3.3 | 16.9 | 4.0 | 16.1 | 2.1 | 16.0 | 34.0 | 122.9 | 226.0 | 4.92 |
| 142 | **S142** | 13.0 | 4.1 | 13.0 | 2.7 | 16.0 | 25.3 | 80.4 | 257.7 | 3.2 | 14.2 | 4.0 | 14.3 | 2.5 | 14.7 | 26.7 | 92.2 | 237.6 | 3.69 |
| 143 | **S143** | 11.0 | 3.7 | 11.0 | 2.1 | 12.7 | 18.0 | 61.8 | 341.7 | 2.5 | 13.5 | 3.5 | 13.5 | 1.7 | 12.7 | 21.3 | 86.2 | 320.5 | 3.45 |
| 144 | **S144** | 13.7 | 4.6 | 13.7 | 2.5 | 15.3 | 26.0 | 132.5 | 342.3 | 5.3 | 14.9 | 4.3 | 14.4 | 2.3 | 15.3 | 26.0 | 137.3 | 345.1 | 5.49 |
| 145 | **S145** | 12.0 | 4.3 | 12.0 | 2.6 | 16.0 | 18.0 | 77.9 | 358.0 | 3.1 | 15.1 | 4.3 | 14.6 | 2.7 | 16.7 | 24.0 | 126.2 | 301.5 | 5.05 |
| 146 | **S146** | 15.0 | 4.1 | 15.0 | 2.2 | 13.3 | 31.3 | 108.7 | 312.3 | 4.3 | 16.1 | 3.7 | 15.6 | 1.8 | 13.3 | 30.3 | 121.5 | 302.2 | 4.86 |
| 147 | **S147** | 11.0 | 3.9 | 11.0 | 2.1 | 14.0 | 21.0 | 82.8 | 352.0 | 3.3 | 12.1 | 3.6 | 11.6 | 1.7 | 13.3 | 21.3 | 84.9 | 300.5 | 3.40 |
| 148 | **S148** | 14.3 | 4.0 | 14.3 | 2.2 | 14.7 | 29.3 | 102.1 | 262.7 | 4.1 | 14.8 | 3.4 | 14.4 | 1.7 | 12.7 | 30.0 | 92.5 | 245.1 | 3.70 |
| 149 | **S149** | 13.7 | 3.9 | 14.3 | 2.2 | 15.3 | 23.0 | 93.7 | 345.3 | 3.7 | 15.7 | 3.8 | 15.2 | 1.9 | 16.0 | 23.3 | 108.2 | 290.3 | 4.33 |
| 150 | **S150** | 15.0 | 4.4 | 15.7 | 2.5 | 16.7 | 28.7 | 116.1 | 303.7 | 4.6 | 18.2 | 4.1 | 18.1 | 2.2 | 14.7 | 33.7 | 135.5 | 274.9 | 5.42 |
| 151 | **K001** | 15.7 | 4.2 | 16.0 | 2.3 | 15.3 | 23.7 | 103.5 | 334.0 | 4.1 | 19.1 | 3.9 | 18.8 | 2.2 | 15.3 | 27.7 | 122.9 | 285.0 | 4.92 |
| 152 | **K002** | 16.7 | 4.0 | 16.7 | 2.3 | 14.7 | 27.0 | 119.6 | 399.7 | 4.8 | 17.9 | 4.1 | 17.9 | 1.9 | 14.7 | 29.0 | 144.7 | 341.7 | 5.79 |

| | | | | | | | | | | | | | | | | | | | |
|---|---|---|---|---|---|---|---|---|---|---|---|---|---|---|---|---|---|---|---|
| 153 | **K003** | 11.0 | 4.1 | 11.0 | 2.1 | 15.3 | 21.0 | 84.3 | 283.7 | 3.4 | 14.7 | 3.9 | 14.3 | 1.8 | 16.0 | 28.3 | 120.0 | 264.4 | 4.80 |
| 154 | **K004** | 13.0 | 4.3 | 13.0 | 2.4 | 17.3 | 26.3 | 96.5 | 264.7 | 3.9 | 15.0 | 4.2 | 14.7 | 2.5 | 18.0 | 28.3 | 112.4 | 222.9 | 4.50 |
| 155 | **K005** | 13.0 | 4.1 | 14.0 | 2.3 | 14.0 | 22.7 | 96.4 | 377.7 | 3.9 | 16.6 | 4.1 | 16.2 | 2.3 | 14.7 | 29.0 | 132.5 | 313.8 | 5.30 |
| 156 | **K006** | 14.3 | 3.8 | 14.3 | 2.3 | 13.3 | 23.3 | 75.8 | 263.3 | 3.0 | 16.9 | 3.4 | 16.3 | 1.8 | 12.7 | 28.7 | 99.3 | 272.1 | 3.97 |
| 157 | **K007** | 14.3 | 3.9 | 14.3 | 2.2 | 12.7 | 29.3 | 106.7 | 312.3 | 4.3 | 17.7 | 3.6 | 18.0 | 1.9 | 12.7 | 31.3 | 121.9 | 309.9 | 4.88 |
| 158 | **K008** | 14.7 | 3.5 | 15.0 | 2.0 | 14.0 | 27.0 | 78.4 | 263.7 | 3.1 | 17.4 | 3.4 | 16.8 | 1.7 | 15.3 | 30.7 | 103.2 | 218.6 | 4.13 |
| 159 | **K009** | 14.7 | 3.9 | 14.7 | 2.3 | 14.0 | 27.0 | 96.0 | 298.0 | 3.8 | 17.7 | 3.7 | 17.2 | 1.9 | 16.0 | 32.3 | 135.2 | 261.4 | 5.41 |
| 160 | **K010** | 13.0 | 3.8 | 13.7 | 2.3 | 14.0 | 21.0 | 72.1 | 293.0 | 2.9 | 17.3 | 3.8 | 17.4 | 2.0 | 14.7 | 28.7 | 116.9 | 279.3 | 4.68 |
| 161 | **K011** | 14.0 | 3.9 | 14.0 | 2.2 | 13.3 | 27.0 | 87.4 | 258.0 | 3.5 | 16.6 | 3.8 | 16.7 | 2.0 | 14.0 | 30.7 | 120.4 | 280.2 | 4.82 |
| 162 | **K012** | 14.7 | 3.9 | 14.7 | 2.3 | 13.3 | 31.3 | 102.0 | 294.0 | 4.1 | 17.5 | 3.7 | 17.3 | 2.1 | 14.7 | 29.3 | 127.6 | 298.7 | 5.11 |
| 163 | **K013** | 12.0 | 4.2 | 12.0 | 2.4 | 15.3 | 24.0 | 93.3 | 310.3 | 3.7 | 14.7 | 4.3 | 14.5 | 2.4 | 18.0 | 26.0 | 115.1 | 245.7 | 4.61 |
| 164 | **K014** | 15.0 | 4.3 | 15.0 | 2.6 | 15.3 | 26.0 | 108.6 | 326.3 | 4.3 | 17.9 | 4.4 | 17.5 | 2.5 | 16.7 | 31.3 | 135.0 | 259.6 | 5.40 |
| 165 | **K015** | 12.3 | 4.4 | 12.3 | 2.5 | 14.7 | 24.7 | 116.8 | 343.3 | 4.7 | 11.9 | 4.3 | 11.1 | 2.5 | 18.7 | 22.3 | 122.3 | 298.2 | 4.89 |
| 166 | **K016** | 15.0 | 3.8 | 15.8 | 2.3 | 14.0 | 23.3 | 84.8 | 328.0 | 3.4 | 18.0 | 3.8 | 18.1 | 2.3 | 16.7 | 27.3 | 130.9 | 288.7 | 5.24 |
| 167 | **K017** | 15.0 | 4.0 | 15.0 | 2.2 | 14.0 | 27.7 | 106.4 | 318.7 | 4.3 | 16.9 | 3.8 | 16.7 | 1.9 | 12.7 | 26.7 | 101.6 | 298.8 | 4.06 |
| 168 | **K018** | 12.3 | 4.4 | 12.7 | 2.3 | 16.7 | 24.0 | 99.8 | 326.7 | 4.0 | 14.8 | 4.0 | 14.5 | 2.2 | 16.0 | 25.7 | 115.7 | 280.9 | 4.63 |
| 169 | **K019** | 15.0 | 4.4 | 15.0 | 2.5 | 14.7 | 30.7 | 127.8 | 330.7 | 5.1 | 16.4 | 4.3 | 16.0 | 2.3 | 13.3 | 26.0 | 123.1 | 357.4 | 4.93 |
| 170 | **K020** | 13.0 | 4.2 | 13.0 | 2.4 | 14.7 | 20.3 | 78.6 | 348.3 | 3.1 | 16.2 | 3.9 | 16.0 | 2.3 | 14.7 | 18.7 | 70.9 | 250.9 | 2.84 |
| 171 | **K021** | 14.0 | 3.7 | 14.0 | 2.1 | 15.3 | 24.3 | 86.1 | 293.3 | 3.4 | 16.5 | 3.4 | 16.0 | 1.8 | 13.3 | 29.7 | 92.7 | 236.6 | 3.71 |
| 172 | **K022** | 11.7 | 3.7 | 11.7 | 2.2 | 14.7 | 21.0 | 57.3 | 235.0 | 2.3 | 12.5 | 3.8 | 12.4 | 2.1 | 16.0 | 22.0 | 81.5 | 234.1 | 3.26 |
| 173 | **K023** | 13.0 | 4.3 | 13.3 | 2.7 | 16.7 | 25.3 | 89.1 | 273.3 | 3.6 | 14.6 | 3.9 | 14.8 | 2.4 | 15.3 | 23.3 | 93.1 | 266.0 | 3.73 |
| 174 | **K024** | 13.7 | 4.4 | 13.7 | 2.4 | 20.0 | 27.3 | 93.6 | 222.0 | 3.7 | 14.1 | 4.3 | 14.4 | 2.3 | 22.0 | 26.0 | 110.4 | 193.0 | 4.42 |
| 175 | **K025** | 15.3 | 4.5 | 15.3 | 2.5 | 18.0 | 28.0 | 118.8 | 262.7 | 4.8 | 16.0 | 4.0 | 16.4 | 2.0 | 16.7 | 26.7 | 104.6 | 235.0 | 4.18 |
| 176 | **K026** | 12.3 | 4.1 | 12.3 | 2.3 | 16.0 | 24.0 | 84.1 | 260.3 | 3.4 | 14.6 | 3.8 | 14.1 | 1.9 | 16.0 | 28.0 | 107.5 | 240.3 | 4.30 |
| 177 | **K027** | 12.0 | 4.0 | 12.0 | 2.3 | 15.3 | 31.0 | 92.3 | 199.0 | 3.7 | 13.5 | 4.3 | 12.7 | 2.0 | 18.7 | 31.3 | 112.8 | 193.2 | 4.51 |
| 178 | **K028** | 13.0 | 4.4 | 13.0 | 2.6 | 16.0 | 27.0 | 100.3 | 261.3 | 4.0 | 14.9 | 4.3 | 14.9 | 2.5 | 14.7 | 28.0 | 101.9 | 244.9 | 4.08 |
| 179 | **K029** | 15.0 | 3.9 | 15.3 | 2.2 | 13.3 | 30.3 | 99.5 | 270.3 | 4.0 | 16.8 | 3.9 | 16.9 | 2.2 | 15.3 | 29.7 | 121.1 | 267.8 | 4.84 |
| 180 | **K030** | 14.0 | 4.2 | 14.0 | 2.1 | 16.0 | 32.0 | 109.5 | 228.0 | 4.4 | 16.2 | 4.2 | 15.9 | 2.1 | 16.7 | 32.0 | 128.4 | 240.3 | 5.14 |
| 181 | **K031** | 12.7 | 4.4 | 14.7 | 2.3 | 14.0 | 30.3 | 123.6 | 327.3 | 4.9 | 15.0 | 4.0 | 14.7 | 2.2 | 14.0 | 30.3 | 120.0 | 281.0 | 4.80 |
| 182 | **K032** | 12.3 | 3.9 | 12.3 | 2.2 | 12.7 | 24.3 | 78.9 | 311.7 | 3.2 | 13.9 | 3.7 | 13.8 | 2.2 | 14.0 | 20.3 | 74.4 | 261.3 | 2.97 |
| 183 | **K033** | 13.0 | 4.1 | 13.3 | 2.4 | 19.3 | 28.0 | 101.8 | 234.3 | 4.1 | 15.8 | 3.9 | 16.1 | 2.2 | 16.7 | 30.3 | 105.8 | 209.7 | 4.23 |
| 184 | **K034** | 11.3 | 4.6 | 11.3 | 2.6 | 17.3 | 22.0 | 90.6 | 286.0 | 3.6 | 13.7 | 4.4 | 13.4 | 2.3 | 17.3 | 26.7 | 113.8 | 246.6 | 4.55 |
| 185 | **K035** | 13.0 | 4.2 | 13.3 | 2.4 | 12.0 | 26.0 | 109.0 | 378.0 | 4.4 | 14.8 | 4.1 | 14.2 | 2.1 | 14.0 | 24.7 | 115.8 | 339.8 | 4.63 |
| 186 | **K036** | 13.7 | 4.2 | 14.0 | 2.4 | 14.7 | 28.7 | 114.7 | 308.0 | 4.6 | 14.2 | 4.0 | 14.0 | 1.9 | 14.0 | 26.3 | 119.5 | 324.9 | 4.78 |
